# Advancing ecological and environmental biology research

**DOI:** 10.1007/s42995-025-00319-4

**Published:** 2025-09-01

**Authors:** Xiao-Hua Zhang, Yun-Wei Dong

**Affiliations:** 1 Frontiers Science Center for Deep Ocean Multispheres and Earth System, and College of Marine Life Sciences, Ocean University of China, Qingdao, 266003 China; 2 Laboratory for Marine Ecology and Environmental Science, Qingdao Marine Science and Technology Center, Qingdao, 266237 China; 3 The Key Laboratory of Mariculture, Ministry of Education, Fisheries College, Ocean University of China, Qingdao, 266003 China; 4 Shandong Key Laboratory of Green Mariculture and Smart Fishery, Fisheries College, Ocean University of China, Qingdao, 266003 China

In recent decades, growing awareness of ecological threats has intensified the urgency of addressing critical global challenges, such as biodiversity collapse, climate disruption, habitat loss, and environmental pollution. These challenges stem from disruptions to Earth’s life-support systems, where living organisms (plants, animals, and microorganisms) interact dynamically through trophic cascades, symbiotic networks, and biogeochemical feedback loops. Together, they form complex ecosystems that regulate climate, cycle nutrients, and sustain planetary health.

Crucially, biodiversity constitutes the foundational architecture of ecosystem health. Its decline degrades food web productivity, diminishes essential services, and risks systemic destabilization. Consequently, comprehensive biodiversity assessment is vital not only for understanding ecosystem structures and functions (e.g., energy flow and biogeochemical cycle) but for evaluating resilience to anthropogenic stressors.

To confront these challenges, critical ecological questions demand urgent exploration: where are the biodiversity hotspots concentrated, how do organisms adapt physiologically and genetically to environmental stressors, what abiotic and biotic factors govern species distribution, abundance, and biodiversity across ecosystems, what mechanisms regulate species interactions, from competition to mutualism, and how do energy and nutrients cycle through ecological networks? Addressing these questions requires a multidisciplinary approach that spans terrestrial, freshwater, and marine environments, encompassing various organizational scales, from molecular to individual, population, and community levels.

Within this framework, marine ecosystems are of paramount importance. Oceans span about 71% of the planet’s surface, sequester about 30% of anthropogenic CO₂ emissions, and generate 50%–85% of atmospheric oxygen via phytoplankton. They harbor extraordinary biodiversity—from coral reefs hosting about 25% of marine species to deep-sea hydrothermal vents sustaining chemosynthetic life—while regulating global biogeochemical cycles. Recent methodological advances, such as genetic data integration, functional trait analysis, and big-data analytics, have revolutionized our understanding of marine biogeography. These insights are essential for predicting how marine systems will respond to anthropogenic pressures, including climate change and over exploitation.

By strategically integrating foundational ecological principles with cutting-edge research, we can dramatically improve predictions of human impacts on natural systems and develop strategies to preserve biodiversity and ecosystem functionality for future generations.

This special issue showcases cutting-edge advances in ecology and environmental biology, with a primary focus on marine ecosystems, through three comprehensive review articles and 13 original research papers. Below, we summarize these contributions.

The origins of marine biodiversity in the Indo-Australian Archipelago (IAA) have long been a significant topic in marine biogeography. Two key hypotheses frame our understanding of the dynamics within the IAA: the "Centers of Hypothesis", which focuses on species accumulation and speciation, and the "Hopping Hotspot Hypothesis", which examines the migration of dynamic hotspots. In our first paper, Huang et al. (2025) provide a synthesis of evidence, ranging from fossil records to phylogeographic data, that supports the new “Dynamic Centers Hypothesis”. This hypothesis suggests that the IAA’s biodiversity hotspot has shifted over geological time. The role of this hotspot in generating and sustaining biodiversity has evolved, with different factors, such as speciation and accumulation, being more influential during distinct historical phases, from Tethyan origins to the current dominance of the Indo-West Pacific. This integrated framework offers a comprehensive understanding of IAA biodiversity. Recent advances in genomics have uncovered extensive cryptic diversity, highlighting the need for multidimensional conservation strategies that incorporate both phylogenetic and functional diversity to protect this hotspot in the face of global change.

Oxygenic photosynthesis on Earth began with cyanobacteria approximately 2.5 billion years ago. Among these, picocyanobacteria (0.5–3 µm in size) play a crucial role in carbon fixation and biogeochemical cycling in marine ecosystems. While their importance in estuarine environments is increasingly recognized, no comprehensive review currently focuses on estuarine picocyanobacteria. In our second paper, Chen (2025) synthesizes two decades of research on estuarine *Synechococcus*, primarily from studies in the temperate Chesapeake Bay. Key findings include the discovery of novel subcluster 5.2 *Synechococcus* lineages, which have advanced our understanding of their ecophysiology, biogeography, genomics, and molecular evolution in Chesapeake Bay and other coastal estuaries. In addition, estuarine picocyanobacteria exhibit enhanced tolerance to fluctuations in temperature, salinity, and heavy metals compared to their coastal and open-ocean counterparts. Furthermore, dynamic seasonal shifts shape the picocyanobacterial community in temperate estuaries. Genomic analyses of these estuarine strains provide new insights into the taxonomy and evolutionary relationships among freshwater, estuarine, and marine unicellular cyanobacteria.

Algae play critical roles in the nutrient cycles of aquatic and atmospheric systems, relying heavily on interactions with co-occurring microbes for growth and survival. Despite their ecological importance, archaea have been largely overlooked in algal research due to their low abundance and the limitations of universal prokaryotic primers in detecting them. This oversight has led to an underestimation of their role in algal symbioses. In our third paper, Lian et al. (2025) summarize recent advances in the diversity of algae-associated archaea and their putative symbiotic relationships, emphasizing the impact of these interactions on biogeochemical cycles. Positive algae-archaea correlations suggest promising avenues for isolating archaeal symbionts. Furthermore, understanding these symbioses could unlock novel applications, including algae-based bioenergy and other biotechnological innovations.

Marine ecosystems are rich reservoirs of diverse microbial communities. However, the majority (> 99%) of these microorganisms remain uncultured and uncharacterized under laboratory conditions. While culture-independent approaches have advanced our understanding of marine microbial assemblages and their metabolic potential, pure cultures remain essential for detailed microbiological studies. Despite increasing efforts to cultivate previously uncultured microorganisms, obtaining pure cultures of ecologically important yet uncultured microbes remains a significant challenge. In our 4th paper, Ahmad et al. (2025) developed a diffusion-based integrative cultivation method using modified low-nutrient media to efficiently isolate previously uncultured bacteria from marine sediments. This innovative approach enabled the successful cultivation of species from rarely cultured phyla, such as *Verrucomicrobiota* and *Balneolota*, outperforming traditional cultivation methods. Remarkably, the application of this novel technique yielded 196 isolates, of which 115 represented previously uncultured taxa, achieving a high novelty ratio of 58%.

Organic matter cycling in the ocean plays a pivotal role in regulating global climate and maintaining ecological balance. Marine organic matter originates primarily from phytoplankton, and its degradation and transformation are driven mainly by heterotrophic prokaryotes. These microorganisms initiate the breakdown of large organic molecules through extracellular enzymes, followed by substrate uptake via specialized transporters. Thus, elucidating the diversity and dynamics of extracellular enzymes and transporters in heterotrophic prokaryotes is essential for understanding organic matter cycling in aquatic ecosystems. In our 5th paper, Liu et al. (2025) employed metagenomics to investigate the functional diversity and temporal dynamics of extracellular enzymes and transporters in coastal waters over a 22-day period. Their findings underscore the critical roles of *Gammaproteobacteria*, *Alphaproteobacteria*, and *Bacteroidota* in organic matter degradation, revealing distinct substrate processing and assimilation strategies among these taxa. Moreover, the study demonstrates a functional linkage between extracellular enzymes and TonB-dependent transporters in marine heterotrophic prokaryotic communities, providing new insights into the mechanistic basis of organic matter cycling.

Methane (CH₄) is a potent greenhouse gas, second only to carbon dioxide in its contribution to global warming. Traditionally, biological methane production has been attributed primarily to strict anaerobic archaea. However, the observed methane supersaturation in oxygen-rich ocean waters—termed the oceanic methane paradox—challenges this notion. Recent studies suggest that microbial degradation of methylphosphonate (MPn) plays a key role in this phenomenon, with evidence pointing to the involvement of *Vibrio* species. In our 6th paper, Yu et al. (2025) investigated MPn-demethylating *Vibrio* strains and the *phn* operons responsible for MPn demethylation. Their findings revealed that MPn-demethylating *Vibrio* species, which may constitute over 28% of all thriving *Vibrio* species, can efficiently convert MPn into methane. The study highlighted the critical role of *phn* operons in this process and uncovered their remarkable genetic diversity. The heterogeneity of *phn* operon types and variations in MPn metabolic capacity among *Vibrio* spp. provide new insights into microbial phosphonate demethylation, advancing our understanding of the methane paradox in aerobic marine environments.

Nitrogen (N) is a key limiting nutrient for primary productivity in marine ecosystems. Anaerobic ammonium oxidation (anammox) serves as a critical pathway for nitrogen removal in estuarine and marine environments. However, the mechanisms driving the formation and maintenance of anammox bacterial communities remain poorly understood. In our 7th paper, Zhang et al. (2025a) investigated the assembly mechanisms of anammox bacterial communities in coastal sediments by analyzing their diversity, community structure, and interspecific interactions across three Chinese estuaries and the South China Sea (SCS). The study revealed high diversity among estuarine anammox bacteria, with ecological drift emerging as the dominant force shaping community assembly. Rare taxa were particularly sensitive to dispersal limitations and environmental selection. Notably, *Candidatus* Scalindua was identified as a keystone genus, while rare species appeared to play a vital role in sustaining the ecological stability of anammox communities in coastal sediments.

Mixotrophy, a strategy combining autotrophic carbon fixation and heterotrophic metabolism, provides a competitive survival advantage for microorganisms in marine ecosystems. *Marinisomatota* (previously recognized as *Marinimicrobia*, Marine Group A, and SAR406) are widespread and highly abundant in marine environments, yet their ecological diversity and metabolic versatility remain poorly characterized. In our 8th paper, Xiang et al. (2025) reconstructed 1588 *Marinisomatota* genomes from global ocean datasets to investigate their metabolic strategies, ecological distribution, and trophic preferences. The study identified three distinct metabolic modes: MS0 (photoautotrophic potential), MS1 (heterotrophic with enhanced glycolytic capacity), and MS2 (heterotrophic without glycolysis), demonstrating the potential for mixotrophic adaptations in *Marinisomatota*. These metabolic strategies likely reflect evolutionary responses to nutrient limitation in oceanic ecosystems.

CO_2_ concentration mechanisms (CCMs) are crucial for the carbon fixation process in marine algae. However, the relative contributions of biophysical and biochemical CCMs to the carbon fixation of marine algae remain unclear. In our 9th paper, Zhang et al. (2025b) observed that inhibiting the biophysical CCM using ethoxyzolamide reduced carbon fixation of the green macroalga *Ulva prolifera* but triggered an increase in biochemical CCM activity, mediated through enhanced cyclic electron flow. This increase compensated for approximately 50% of the total carbon fixation. Conversely, when the biochemical CCM was inhibited with 3-mercaptopicolinic acid, the reduction in carbon fixation was fully compensated (100%) by an increase in biophysical CCM activity. These results indicate that biophysical CCMs are the dominant mechanism for carbon fixation in *U. prolifera*, while biochemical CCMs play a supportive role. The findings reveal a complementary coordination mechanism between biophysical and biochemical CCMs in *U. prolifera*. This synergy optimizes photosynthetic efficiency under varying conditions and is a key physiological adaptation that contributes to the alga’s ability to form large blooms.

Understanding how zooplankton communities respond to environmental changes and identifying the key drivers of their dynamics are essential for the sustainable management of aquaculture ponds. In our 10th paper, Mao et al. (2025) examined three types of aquaculture ponds—crab, crayfish, and fish ponds—with over 10 years of historical data. They analyzed 27 environmental factors that could potentially influence zooplankton dynamics throughout the year. The study clarified community compositions and identified the environmental drivers of biodiversity. It was found that Rotifera dominated all pond types, followed by Protista, Cladocera, and Copepoda. The dominant species included *Polyarthra vulgaris*, *Anuraeopsis fissa*, and *Trichocerca pusilla*. Alpha diversity of zooplankton was influenced by distinct environmental factors specific to each pond type, and antibiotics had a significant effect only on the fish ponds. Deterministic processes, primarily driven by temperature and ammonia nitrogen, governed community assembly. These findings provide critical insights to guide aquaculture management practices. Optimizing zooplankton dynamics based on key drivers identified can enhance ecosystem stability and productivity, thereby promoting the sustainable development of the aquaculture industry.

Surviving extreme cold events is essential for the population maintenance of intertidal species in the context of climate change. It is crucial to understand how microhabitat heterogeneity influences body temperature and metabolomic responses of intertidal mollusks during these extreme low-temperature events. In our 11th paper, Zhang et al. (2025c) found that the body temperature of intertidal mollusks was primarily influenced by microhabitat type, substrate temperature, air temperature, wind speed, and light intensity. Additionally, mollusks exhibited microhabitat-specific metabolomic responses that included cellular stress response, energy metabolism, immune response, nucleotide metabolism, and osmoregulation. The levels of these metabolites were significantly correlated with microclimate variables, such as substrate temperature, wind speed, and body temperature. These findings demonstrate that microhabitat heterogeneity plays a critical role in determining cold-stress resilience in intertidal species. This insight is vital for predicting how species will adapt to the increasing frequency of extreme cold events.

Habitat fragmentation poses a grand challenge for population maintenance, making it crucial to understand how it influences thermal tolerance in the dimorphic ant species *Pheidole nodus* across edge and interior habitats, different seasons, and various castes within a fragmented island system. In our 12th paper, Zhao et al. (2025) discovered that edge habitats were 1–3 °C warmer than interior habitats during the hot season, and edge populations on smaller islands exhibited different thermal limits compared to those in interior populations. Additionally, minor workers consistently demonstrated a higher upper thermal limit (CTmax) and a lower critical thermal minimum (CTmin) than major workers (soldiers). This study highlights how thermal tolerance is affected by factors such as island area, habitat type, seasonality, and social caste and provides a framework for incorporating thermal physiology into research on habitat fragmentation.

Assessing the fine-scale population structure and genetic diversity of queen snapper (*Etelis oculatus*) in the waters of Puerto Rico is crucial for enhancing conservation management. It is important to clarify whether queen snapper exhibit fine-scale genetic population structure across different sampling sites in Puerto Rico and to determine its level of genetic diversity in this region. In our 13th paper, González-García et al. (2025) found that there was low genetic diversity across sampling sites, and no significant differentiation was detected. Principal component analysis and clustering further confirmed the absence of fine-scale subpopulations among the sites. This study provides the first genetic baseline for queen snapper in the waters of Puerto Rico, revealing high connectivity and low genetic diversity. As deep-sea fishing pressure increases, these findings underscore the urgent need for biological and ecological research to inform science-based management and conservation of this commercially important species.

The silver pomfret *Pampus argenteus* is one of the most commercially important fish species in the West Pacific. It is important to investigate whether the spawning behavior and habitat preferences among coastal stocks of *P. argenteus* along China’s coast reflect underlying genetic divergence and local adaptation. In our 14th paper, Wei et al. (2025) found that there was no hierarchical genetic differentiation among the six stocks of *P. argenteus* along China’s coast. However, 21 genomic regions exhibited significant isolation by distance and environment. Some genes within these divergent windows were functionally linked to environmental adaptation and behavioral variation. The study reveals that local adaptation in *P. argenteus* occurs despite high gene flow, driven by selection on specific genomic regions that respond to environmental gradients such as temperature and photoperiod. This result presents a key genomic basis for understanding the stock-specific management needs under environmental change.

Integrating physiological traits into species distribution models (SDMs) is a novel and important approach to improving predictions of invasion risk. In our 15th paper, Oskyrko et al. (2025) aim to incorporate temperature-dependent sex determination (TSD) into SDMs. This integration helps forecast current and future suitable habitats for the invasive freshwater pond slider turtle, *Trachemys scripta*, and assess which regions are at the highest risk of invasion due to climate change. The study found that suitable habitats for the turtle exist in 136 countries worldwide, with habitat loss projected to occur in 78 to 93 countries under various future scenarios. This research represents the first integration of sex ratio constraints into SDMs specifically for the invasion ecology of this turtle species. It demonstrates that physiology-trait hybrid SDMs are crucial for developing targeted strategies to control globally invasive reptiles in the face of climate change.

Physiological adaptation is a crucial strategy for animals surviving temperature stress. Understanding the metabolic and mitochondrial responses to prolonged heat exposure is essential for identifying the mechanisms that enable high thermal adaptability. In our 16th paper, Pan et al. (2025) aimed to investigate the physiological mechanisms by which heat acclimation remodels mitochondrial membranes and alters metabolic function to reduce energy expenditure while preserving acute cold response capabilities. The researchers found that heat-acclimated gerbils exhibited lower basal metabolic rates but maintained their non-shivering thermogenesis capacity. Lipidomics analyses revealed increased ratios of monounsaturated to polyunsaturated fatty acids in the mitochondrial membranes, which correlated with reduced metabolic rates. Additionally, cytochrome C oxidase activity increased in brown adipose tissue, likely due to greater membrane unsaturation. These results suggest that mitochondrial membrane plasticity serves as a key adaptation strategy, allowing gerbils to minimize energy expenditure during heat acclimation. Furthermore, the composition of membrane lipids fine-tunes metabolic efficiency and thermal flexibility.

The 16 articles in this special issue showcase significant advances in ecology and environmental biology, particularly in marine systems. We hope these contributions inspire further research to address pressing ecological challenges and inform conservation strategies.
